# Trilaciclib for prophylaxis of chemotherapy-induced myelosuppression in solid tumor patients: a systematic review and meta-analysis

**DOI:** 10.3389/fphar.2026.1754502

**Published:** 2026-04-08

**Authors:** Ting Yang, Pengjie Yang, Danyang Meng, Yong Zhu, Benben Zhu

**Affiliations:** 1 College of Pharmacy, Inner Mongolia Medical University, Hohhot, China; 2 Department of Thoracic Surgery, Peking University Cancer Hospital (Inner Mongolia Campus)/Affiliated Cancer Hospital of Inner Mongolia Medical University, Hohhot, China; 3 Department of Pathology, Peking University Cancer Hospital (Inner Mongolia Campus)/Affiliated Cancer Hospital of Inner Mongolia Medical University, Hohhot, China; 4 Department of Surgery of Bone and Soft Tissue Tumors, Peking University Cancer Hospital (Inner Mongolia Campus)/Affiliated Cancer Hospital of Inner Mongolia Medical University, Hohhot, China; 5 Department of Pharmacy, Peking University Cancer Hospital (Inner Mongolia Campus)/Affiliated Cancer Hospital of Inner Mongolia Medical University, Hohhot, China

**Keywords:** chemotherapy, chemotherapy-induced myelosuppression (CIM), randomized controlled trial (RCT), solid tumors, trilaciclib

## Abstract

**Objective:**

To systematically evaluate the clinical benefit and safety of trilaciclib in solid-tumor patients receiving chemotherapy and to inform clinical practice.

**Methods:**

Following PRISMA guidelines and registered at PROSPERO (CRD420251053232), we searched PubMed, Embase and two other databases from inception to June 2025. Six randomized controlled trials enrolling 726 patients were included. Meta-analyses were performed with Review Manager 5.4.

**Results:**

Trilaciclib significantly reduced the incidence of severe neutropenia (SN) and febrile neutropenia (FN), shortened SN duration, and decreased the need for erythropoiesis-stimulating agents (ESAs), granulocyte colony-stimulating factor (G-CSF) and red-blood-cell (RBC) transfusions while lowering anemia rates. These benefits were not accompanied by increased risks of nausea, vomiting or fatigue. Progression-free survival (PFS) was significantly prolonged, whereas overall survival (OS) remained unchanged; patients aged ≥65 years and those enrolled in U.S. trials derived the greatest benefit. Limitations include the small number of RCTs, heterogeneous chemotherapy regimens, potential publication bias and short follow-up in some studies.

**Conclusion:**

Trilaciclib effectively prevents chemotherapy-induced myelosuppression in solid-tumor patients and can guide clinical use, but further well-designed studies are warranted to consolidate its efficacy and safety profile.

**Systematic Review Registration:**

https://www.crd.york.ac.uk/PROSPERO/, identifier CRD420251053232URL.

## 1 Introduction

Chemotherapy remains a cornerstone of cancer treatment, yet one of its most frequent toxicities is chemotherapy-induced myelosuppression (CIM) (Sharma et al., 2024). CIM manifests as a sequential decline in white blood cells, erythrocytes and platelets, predisposing patients to infection, anemia and bleeding (Crawford et al., 2024). Beyond immediate morbidity, myelosuppression may force dose delays or outright discontinuation, compromising anti-tumour efficacy (Utomo et al., 2024). In extensive-stage small-cell lung cancer (ES-SCLC), the burden is particularly stark: >60% of patients experience at least one grade ≥3 cytopenia during any treatment cycle, with grade ≥3 neutropenia reported in 42.7%, anemia in 37.2% and thrombocytopenia in 36.1% (Hart et al., 2023). Current interventions—granulocyte colony-stimulating factor (G-CSF) and erythropoiesis-stimulating agents—address only single lineage toxicities and leave thrombocytopenia and anemia largely unmet. A safe, convenient, multilineage myeloprotective agent is therefore an urgent clinical unmet need.

Trilaciclib is a first-in-class, reversible, selective inhibitor of cyclin-dependent kinases four and 6 (CDK4/6). In February 2021 it became the first agent licensed by the FDA specifically for myeloprotection. When administered intravenously before chemotherapy, trilaciclib transiently arrests CDK4/6-dependent haematopoietic stem and progenitor cells (HSPCs) and lymphocytes in the G1 phase of the cell cycle, shielding them from chemotherapy-induced DNA damage and apoptosis (He et al., 2017). Randomised controlled trials conducted largely in small-cell lung cancer have shown that this strategy significantly reduces the incidence of grade 3/4 neutropenia, anemia and thrombocytopenia, lowers the need for G-CSF and red-cell transfusions, and delays the onset of dose-limiting toxicities (Li et al., 2023; Ding et al., 2025). To date, however, these data remain fragmented; a systematic synthesis of the available RCT evidence is lacking.

This study compared the clinical benefits and safety of trilaciclib in patients receiving therapeutic chemotherapy regimens, providing evidence for clinical decision-making.

## 2 Methods

This systematic review and meta-analysis was conducted in accordance with the Preferred Reporting Items for Systematic Reviews and Meta-Analyses (PRISMA) guideline (Supplementary Material S1), and the protocol was registered with PROSPERO (registration No. CRD420251053232).

### 2.1 Search strategy

Two reviewers independently searched PubMed, Embase, the Cochrane Library and Web of Science for articles evaluating the efficacy and safety of trilaciclib for preventing chemotherapy-induced myelosuppression, covering records from inception to June 2025 without language or date restrictions. The following terms were used: (“trilaciclib” OR “Cosela” OR “G1T28”) AND (“chemotherapy-induced myelosuppression” OR “myelosuppression” OR “neutropenia” OR “bone marrow suppression”) AND (“chemotherapy” OR “antineoplastic agents”) AND (“solid tumor” OR “solid tumour”). Search strategies were adapted for each database using appropriate controlled vocabulary, including MeSH terms in PubMed and Emtree terms in Embase. Reference lists of all relevant publications were hand-searched to identify additional studies potentially meeting the inclusion and exclusion criteria.

### 2.2 Eligibility criteria

Eligibility criteria were defined according to the PICOS framework. The population (P) included adult patients with solid tumors receiving chemotherapy. The intervention (I) was trilaciclib administered prior to chemotherapy. The comparison (C) was placebo or chemotherapy alone without trilaciclib. The outcomes (O) included hematologic toxicity (e.g., severe neutropenia, febrile neutropenia), supportive care use (e.g., G-CSF, ESAs, transfusions), survival outcomes (OS and PFS), and safety outcomes. The study design (S) was restricted to randomized controlled trials. Inclusion criteria: (1) Adults with solid tumors scheduled to receive myelosuppressive chemotherapy who were assigned to trilaciclib for myeloprophylaxis. (2) Comparison of trilaciclib plus chemotherapy versus chemotherapy alone (placebo or no additional intervention). (3) Report of ≥1 predefined outcome: incidence of severe neutropenia (SN) or febrile neutropenia (FN); use of erythropoiesis-stimulating agents (ESAs), granulocyte colony-stimulating factors (G-CSFs), red-blood-cell (RBC) or platelet transfusions; duration of severe neutropenia (DSN); occurrence of anemia, diarrhoea, nausea, vomiting or other adverse events; overall survival (OS, time from randomization to death from any cause) or progression-free survival (PFS, time from randomization to documented progression or death). In all included RCTs, prophylactic administration of ESAs and G-CSF was strictly prohibited during Cycle 1 to avoid interfering with the assessment of trilaciclib’s intrinsic myeloprotective efficacy. However, therapeutic use of ESAs and G-CSF (i.e., administration to manage established myelosuppression, such as grade ≥3 neutropenia or symptomatic anemia) was permitted in all treatment cycles (including Cycle 1) per clinical practice guidelines. Exclusion criteria: (1) Insufficient data or unobtainable full text. (2) Duplicate publications. (3) Systematic reviews, case reports, letters, conference abstracts, commentaries or unpublished data.

### 2.3 Data extraction

Records retrieved from the databases were imported into EndNote, deduplicated, and then screened independently by two reviewers against the eligibility criteria. Discrepancies were resolved through discussion with a third investigator. For each included trial the following information was extracted: Baseline characteristics: first author, year of publication, study design; Study characteristics: sample size, patient population, chemotherapy regimen, clinical outcomes, and adverse-event risk.

### 2.4 Risk-of-bias assessment

Because all eligible studies were randomized controlled trials, methodological quality and risk of bias were appraised independently by two reviewers with the Cochrane RoB-2 tool. The seven domains evaluated were: random sequence generation, allocation concealment, blinding of participants and personnel, blinding of outcome assessment, incomplete outcome data, selective reporting, and other sources of bias. Trials judged as low risk across all seven domains were rated as high quality (grade A); those with some concerns were rated as moderate quality (grade B); trials with one or more domains at high risk were classified as low quality (grade C). To assess the robustness of the pooled results, sensitivity analyses were conducted by excluding studies judged to be at high risk of bias in key domains.

### 2.5 Statistical analysis

A meta-analysis was performed using Review Manager 5.4 software. For survival data (progression-free survival [PFS] and overall survival [OS]), effect sizes were evaluated using hazard ratios (HR) with 95% confidence intervals (95% CI). For categorical data, effect sizes were assessed using odds ratios (OR) with 95% CI. When substantial heterogeneity was present (*I* ^2^ > 50%), a random-effects model (REM) was applied to account for between-study variability. A fixed-effects model (FEM) was used when heterogeneity was low or moderate (*I* ^2^ ≤ 50%). Disease-based subgroup analyses were not performed because the number of trials in non-SCLC populations was insufficient to support reliable subgroup comparisons. Sensitivity analyses were conducted to evaluate the robustness of pooled estimates. Statistical heterogeneity across studies was evaluated using the P-value and *I* ^2^ statistic: a P-value <0.05 indicated statistically significant heterogeneity.

## 3 Results

### 3.1 Literature search results

**FIGURE 1:**
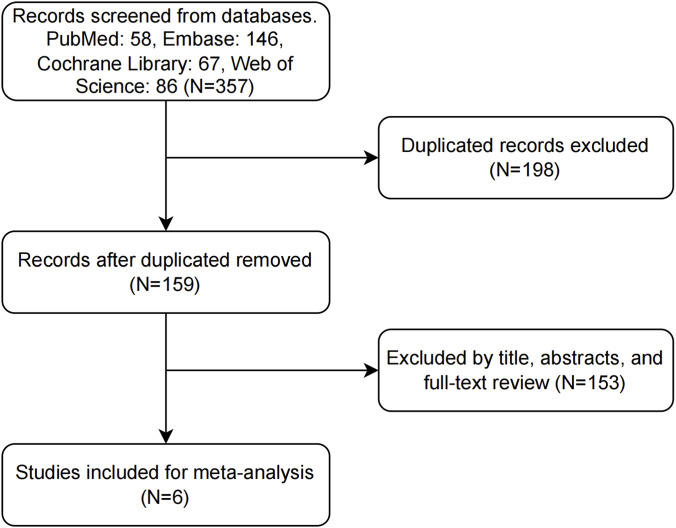
PRISMA flow diagram the search strategy and results.

A systematic search was conducted across English databases, with the search period restricted from the establishment of each database to June 2025. A total of 357 relevant studies were retrieved, including 58 from PubMed, 146 from Embase, 67 from the Cochrane Library, and 86 from Web of Science. After removing 198 duplicate studies, 153 articles were further excluded through screening—including case reports, commentaries, letters, reviews, and studies from which outcome indicators could not be extracted. Finally, six studies were included in the meta-analysis, all of which were randomized controlled trials (RCTs) (Figure 1).

### 3.2 Basic characteristics of included studies

A total of 726 patients were enrolled across the 6 included RCTs, with 383 patients receiving trilaciclib and 343 patients not receiving trilaciclib. The six studies adopted different therapeutic regimens, namely gemcitabine/carboplatin (G/P), etoposide/carboplatin/atezolizumab (E/P/A), etoposide/carboplatin (E/P), topotecan, and fluorouracil/leucovorin/oxaliplatin/irinotecan plus bevacizumab (Table 1). Among these studies, four focused on patients with small-cell lung cancer (SCLC), one on patients with colorectal cancer (CRC), and one on patients with triple-negative breast cancer (TNBC). Prophylactic administration of erythropoiesis-stimulating agents (ESAs) or granulocyte colony-stimulating factor (G-CSF) was prohibited during Cycle one to avoid interfering with the results, while therapeutic use of ESAs or G-CSF was permitted in all cycles. All trials were designed and conducted in accordance with the Declaration of Helsinki and the Good Clinical Practice (GCP) Guidelines of the International Council for Harmonisation (ICH).

**TABLE 1:** Characteristics of the included studies.

Authors	Published year	Treatment modality		Age (years), median		Gender (n)		Number of patients	
		Experimental	Control	Experimental	Control	Experimental	Control	Experimental	Control
Heinz-Josef Lenz	2025	Trilaciclib prior to FOLFOXIRI/bevacizumab	Placebo prior to FOLFOXIRI/bevacizumab	58 (26–81)	55 (30–79)	Male 94, Female 55	Male 91, Female 56	149	147
Ying Cheng	2024	Trilaciclib	Placenbo	63 (45–77)	60.5 (39–76)	Male 33, Female 8	Male 35, Female 7	41	42
Lowell L. Hart	2021	Trilaciclib prior to topotecan	Placebo prior to topotecan	62 (47–77)	64 (47–82)	Male 22, Female 10	Male 12, Female 17	32	29
Davey Daniel	2020	Trilaciclib prior to E/P/A	Placebo prior to E/P/A	65 (45–81)	64 (46–83)	Male 41, Female13	Male 34, Female19	54	53
J. M. Weiss	2019	E/P + trilaciclib	E/P + placebo	64 (49–82)	66 (39–86)	Male 27, Female 11	Male 27, Female 12	39	38
Antoinette R. Tan	2019	G/P plus trilaciclib (D1+D8)	G/P plus placebo	55 (47–66)	55 (43–64)	Male 1, Female 32	Male 0, Female 34	33	34
		G/P plus trilaciclib (D2+D9)		58 (49–65)		Male 0, Female 35		35	

Antoinette R. Tan et al. (2019) was a multi-arm randomized controlled trial with two independent experimental arms (Trilaciclib administered on Days 1 + 8 or Days 2 + 9) sharing the same placebo control arm. Data for the control arm are presented once to avoid redundancy. Sex distribution is reported as absolute numbers (n) for males and females to ensure transparency.

### 3.3 Quality assessment of included studies

**FIGURE 2:**
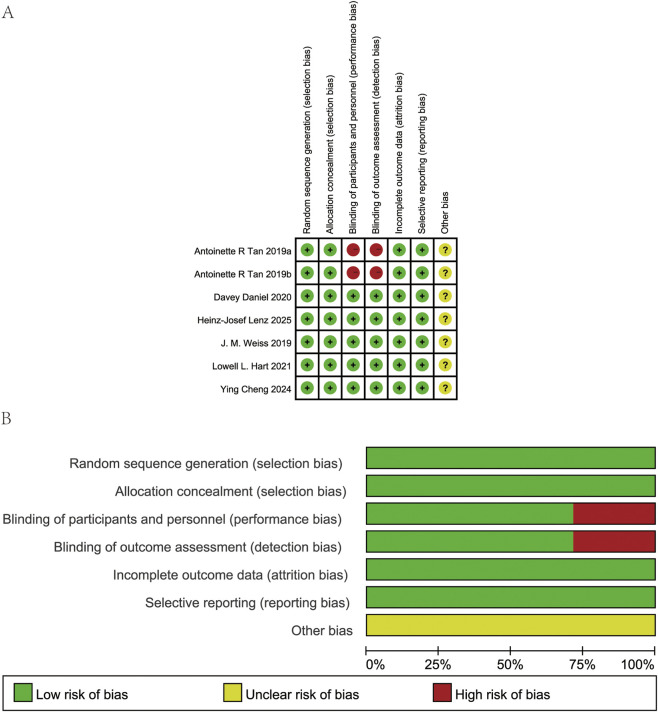
Risk of bias assessment at the study level. **(A)** Risk of bias graph: review authors’ judgment about each risk of bias item presented as percentages across all included full reported studies. **(B)** Risk of bias summary: review authors’ judgment about each risk of bias item for each included study.

The Cochrane Collaboration’s Risk of Bias tool was used to assess the quality of the included studies. The results indicated that most studies had a low risk of bias, and the overall quality of the included studies was good. However, Tan’s study showed a high risk of bias in the implementation of blinding of participants and outcome assessment. Therefore, the impact of this study should be taken into account when interpreting the results subsequently (Figure 2).

### 3.4 Meta-analysis results

#### 3.4.1 Efficacy

**FIGURE 3:**
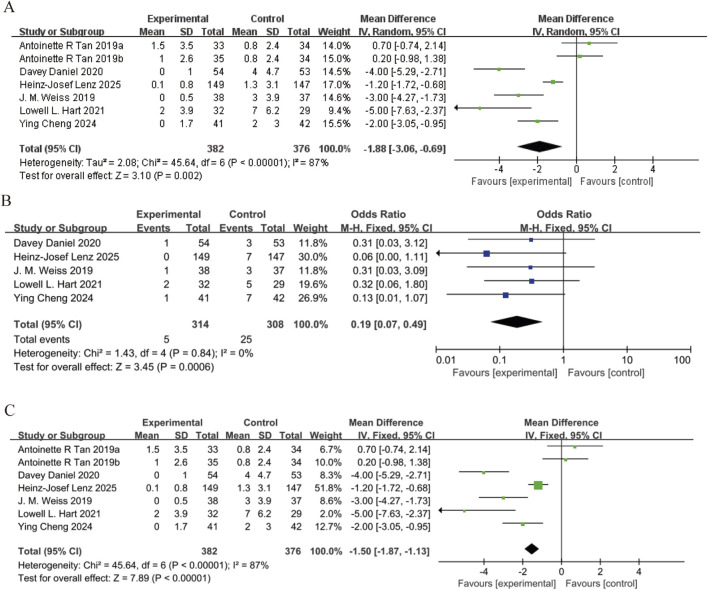
Forest plots of odds ratio (OR) for outcomes. **(A)** Severe neutropenia. **(B)** Febrile neutropenia. **(C)** Duration of neutropenia. Analyzed using the inverse variance (IV) fixed-effect model.

**FIGURE 4:**
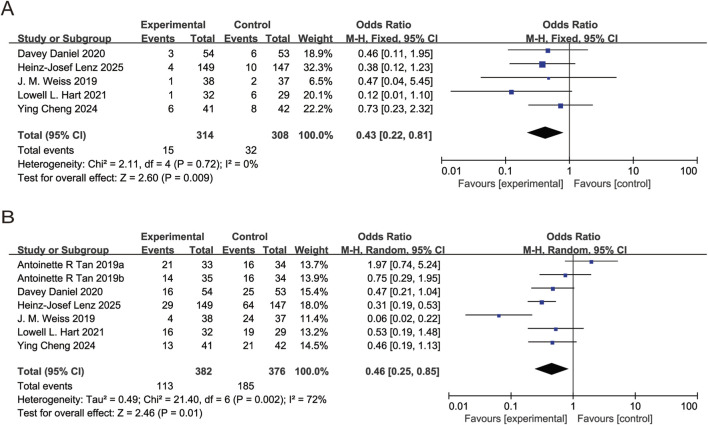
Forest plots of odds ratio (OR) for outcomes. **(A)** Erythropoiesis-Stimulating Agent (ESA) infusion. **(B)** Granulocyte Colony-Stimulating Factor (G-CSF) infusion. Analyzed using the Mantel-Haenszel (M–H) fixed-effect model.

All six studies reported the incidence of neutropenia (SN). With an *I* ^2^ statistic of 81%, a random-effects model was applied. The pooled odds ratio (OR) for the incidence of SN was 0.2 (95% confidence interval [95% CI]: 0.13 to 0.31, P < 0.05), indicating that the trilaciclib group significantly reduced the incidence of SN (Figure 3A). Five studies reported the incidence of febrile neutropenia (FN). With an *I* ^2^ statistic of 0%, a fixed-effects model was used. The pooled OR for the incidence of FN was 0.19 (95% CI: 0.07 to 0.49, P < 0.05), demonstrating a significant reduction in the incidence of FN in the trilaciclib group (Figure 3B). All six studies reported the duration of severe neutropenia (DSN). With an *I* ^2^ statistic of 87%, a random-effects model was employed. The pooled mean difference (MD) for DSN was −1.5 (95% CI: −1.87 to −1.13, P < 0.05), suggesting that the trilaciclib group significantly shortened the duration of severe neutropenia (Figure 3C). Five studies reported ESAs administration. *I* ^2^ = 0%, FEM used. Pooled OR for ESAs administration = 0.43 (95% CI: 0.22 to 0.81, P < 0.05), indicating trilaciclib group significantly reduced ESAs use need (Figure 4A). All six studies reported G-CSF administration. *I* ^2^ = 72%, REM employed. Pooled OR for G-CSF administration = 0.43 (95% CI: 0.32 to 0.58, P < 0.05), demonstrating trilaciclib group significantly decreased G-CSF use requirement (Figure 4B).

**FIGURE 5:**
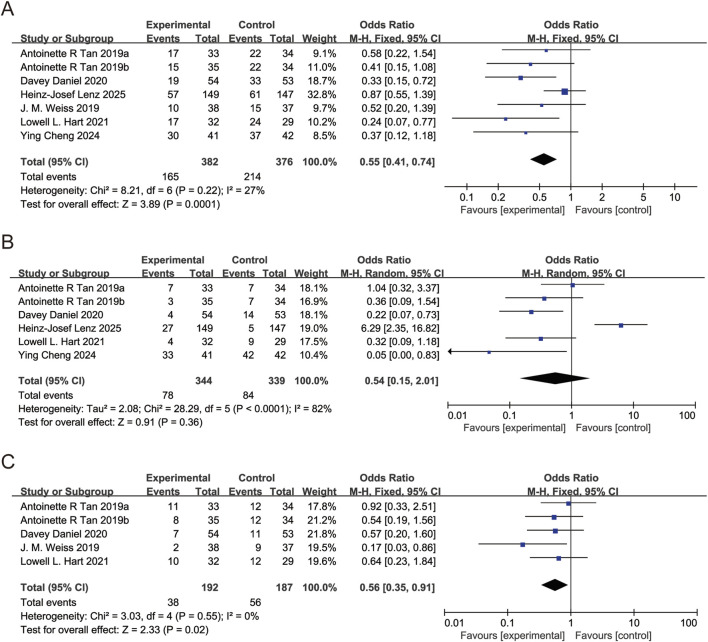
Forest plots of odds ratio (OR) for outcomes. **(A)** Anemia. **(B)** Leukopenia. **(C)** Red blood cell transfusion. Analyzed using the Mantel-Haenszel (M-H) fixed-effect model.

All six studies reported anemia incidence. *I* ^2^ = 27%, FEM used. Pooled OR for anemia incidence = 0.55 (95% CI: 0.41 to 0.74, P < 0.05), indicating trilaciclib group significantly reduced anemia incidence (Figure 5A). Five studies reported leukopenia incidence. *I* ^2^ = 82%, REM used. Pooled OR for leukopenia incidence = 0.87 (95% CI: 0.57 to 1.33, P > 0.05), showing no significant difference in leukopenia incidence between trilaciclib and placebo groups (Figure 5B). Four studies reported RBC transfusion. *I* ^2^ = 0%, FEM used. Pooled OR for RBC transfusion = 0.56 (95% CI: 0.35 to 0.91, P < 0.05), demonstrating trilaciclib group significantly decreased RBC transfusion (Figure 5C).

**FIGURE 6:**
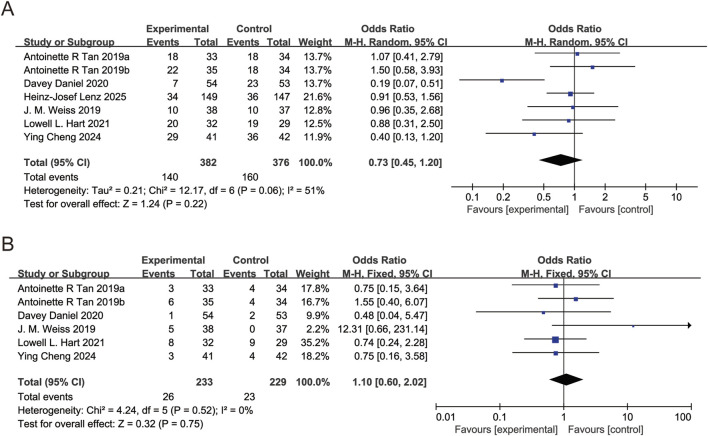
Forest plots of odds ratio (OR) for outcomes. **(A)** Thrombocytopenia. **(B)** Platelet transfusion. Analyzed using the Mantel-Haenszel (M-H) fixed-effect model.

All six studies reported thrombocytopenia incidence. *I* ^2^ = 51%, REM used. Pooled OR for thrombocytopenia incidence = 0.75 (95% CI: 0.55, 1.03, P > 0.05), showing no significant difference in thrombocytopenia incidence between trilaciclib and placebo groups (Figure 6A). Five studies reported platelet transfusion. *I* ^2^ = 0%, FEM used. Pooled OR for platelet transfusion = 1.10 (95% CI: 0.60, 2.02, P > 0.05), demonstrating no significant difference in platelet transfusion between trilaciclib and placebo groups (Figure 6B).

#### 3.4.2 Safety

**FIGURE 7:**
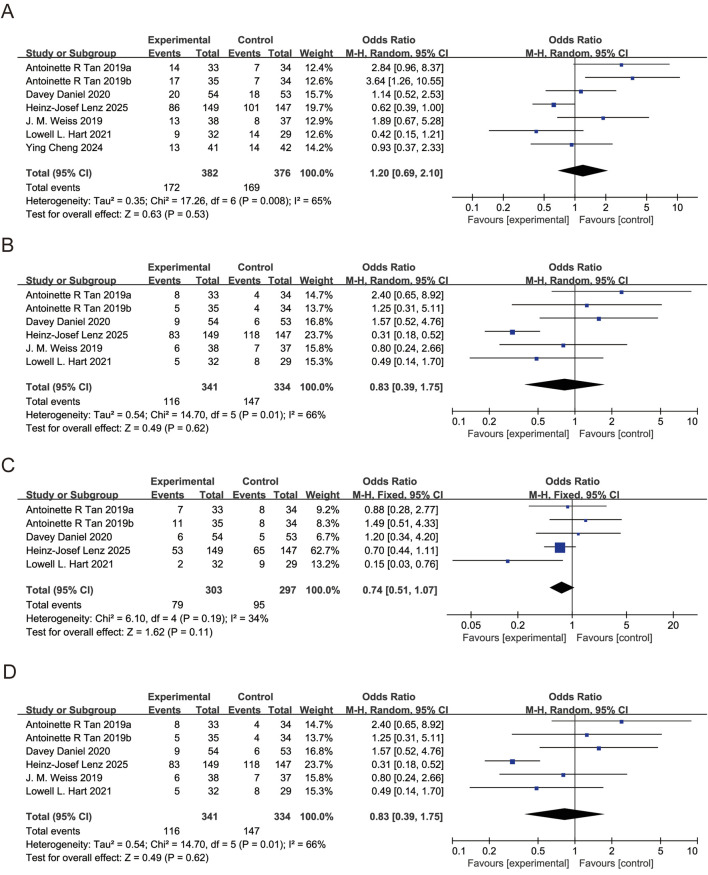
Forest plots of odds ratio (OR) for outcomes. **(A)** Nausea. **(B)** Diarrhea. **(C)** Vomiting. **(D)** Fatigue, all analyzed using the Mantel-Haenszel (M-H) fixed-effect model.

Five studies reported diarrhea incidence. *I* ^2^ = 66%, REM used. Pooled OR for diarrhea incidence = 0.57 (95% CI: 0.40, 0.82, P < 0.05), indicating trilaciclib group significantly reduced diarrhea incidence (Figure 7B). The effects of trilaciclib on nausea, vomiting, and fatigue are shown in Figures 7A,C,D, respectively. For nausea (*I* ^2^ = 65%) and fatigue (*I* ^2^ = 70%), random-effects models were used, and no significant differences were observed between the trilaciclib and control groups (nausea: OR = 1.00, 95% CI: 0.75 to 1.35, P > 0.05; fatigue: OR = 1.08, 95% CI: 0.78 to 1.48, P > 0.05). For vomiting, heterogeneity was low (OR = 0.74, 95% CI = 0.51 to 1.07, *I* ^2^ = 34%, P > 0.05); therefore, a fixed-effect model was applied, showing no significant difference between the two groups (OR = 0.74, 95% CI: 0.51 to 1.07, P > 0.05).

OS and PFS were reported in 5 studies each. OS using REM, PFS using FEM, the pooled HR for OS was 0.87 (95% CI: 0.72, 1.05, *I* ^2^ = 73%, P > 0.05), indicating trilaciclib group did not improve patients’ OS; the pooled HR for PFS was 0.77 (95% CI: 0.64, 0.92, *I* ^2^ = 0%, P < 0.05), demonstrating trilaciclib group improved patients’ prognosis and prolonged PFS (Supplementary Figure S1).

### 3.5 Subgroup analysis

Patients were divided into two age subgroups: <65 years and ≥65 years. Results showed that patients ≥65 years achieved greater benefits (HR = 0.54, 95% CI = 0.34 to 0.84, P < 0.05), while no significant difference was observed in patients <65 years (HR = 0.83, 95% CI = 0.56 to 1.22, P > 0.05) (Supplementary Figure S2A). Further subgroup analysis was performed by ethnicity, dividing patients into Americans and non-Americans. Results demonstrated that American patients had greater benefits (HR = 0.54, 95% CI = 0.36 to 0.82, P < 0.05), whereas non-American patients showed no significant difference (HR = 0.62, 95% CI = 0.46 to 0.84, P > 0.05) (Supplementary Figure S2B).

### 3.6 Sensitivity analysis

Due to significant heterogeneity observed in several outcomes, sensitivity analyses were performed by sequentially excluding individual studies. After exclusion of Antoinette R. Tan et al. (2019), heterogeneity was substantially reduced for SN incidence (*I* ^2^ = 30%), G-CSF administration (*I* ^2^ = 55%), and OS (*I* ^2^ = 45%). Importantly, the pooled estimates for SN incidence (OR = 0.08, 95% CI: 0.05 to 0.15, P < 0.05) and G-CSF administration (OR = 0.33, 95% CI: 0.23 to 0.46, P < 0.05) remained statistically significant, while no significant difference in OS was observed between the trilaciclib and placebo groups (OR = 0.99, 95% CI: 0.81 to 1.22, P > 0.05) (Supplementary Figure S3A,B,D). After exclusion of Heinz-Josef Lenz et al. (2025), heterogeneity in leukopenia incidence decreased (*I* ^2^ = 31%), and the pooled result changed, showing a statistically significant reduction in leukopenia incidence in the trilaciclib group (OR = 0.33, 95% CI: 0.19 to 0.60, P < 0.05) (Supplementary Figure S3C). Similarly, exclusion of Antoinette R. Tan et al. (2019) for nausea incidence and Heinz-Josef Lenz et al. (2025) for diarrhea incidence markedly reduced heterogeneity (*I* ^2^ = 34% and 0%, respectively). While the pooled estimate for nausea incidence remained non-significant (OR = 0.79, 95% CI: 0.57 to 1.10, P > 0.05), the previously observed reduction in diarrhea incidence was no longer statistically significant after exclusion (OR = 1.13, 95% CI: 0.65 to 1.94, P > 0.05) (Supplementary Figure S4A,B). Exclusion of Davey Daniel et al. (2020) for fatigue incidence also reduced heterogeneity (*I* ^2^ = 16%), with no significant difference observed between groups (OR = 1.36, 95% CI: 0.96 to 1.91, P > 0.05) (Supplementary Figure S4C). Overall, sensitivity analyses indicated that while most primary hematologic outcomes were robust, the effect sizes for certain safety outcomes were attenuated after exclusion of studies with higher risk of bias, suggesting that methodological limitations may partially influence the pooled estimates.

## 4 Discussion

Chemotherapy-induced myelosuppression (CIM) is a common dose-limiting adverse reaction in cancer treatment with extensive and profound impacts. Currently, trilaciclib is clinically used to reduce CIM and improve patients’ quality of life. However, the clinical and safety outcomes of trilaciclib have not been fully evaluated. Therefore, this study systematically analyzed and explored trilaciclib. In terms of efficacy: Patients who received trilaciclib before chemotherapy had lower incidences of SN, FN, and DSN than the control group. The administration frequencies of ESAs, G-CSF, and RBC were also lower in the trilaciclib group, as were the incidences of anemia and leukopenia. No significant differences were observed in the incidence of thrombocytopenia or platelet transfusion between the trilaciclib group and the control group. Regarding OS and PFS: Trilaciclib significantly prolonged patients’ PFS; however, there was no statistical difference in OS benefit between the trilaciclib group and the placebo group. In terms of safety: No significant differences were found in the incidences of nausea, vomiting, diarrhea, and fatigue between patients who received trilaciclib before chemotherapy and the control group.

This study found that trilaciclib can significantly reduce the incidence of chemotherapy-related neutropenia in chemotherapy patients and shorten the duration of neutropenia. Chemotherapeutic agents exert antitumor effects by damaging DNA, inhibiting nucleic acid synthesis, or blocking cell division; these targeted mechanisms also act on rapidly proliferating hematopoietic precursor cells in the bone marrow, leading to cell cycle arrest, oxidative stress, disrupted inflammatory signaling, and stromal damage, which ultimately manifest as reductions in neutrophils, red blood cells, and platelets (Grabowski et al., 2021; Bukowski et al., 2020; Yi et al., 2022; Yu et al., 2023). Currently, therapeutic approaches for myelosuppression mainly target a single blood cell lineage, making it difficult to simultaneously restore all blood cell lines. Additionally, these treatments require a long administration duration, resulting in delayed efficacy and adverse reactions related to medications and blood transfusions (Liu et al., 2023). Repeated mobilization of hematopoietic stem cells after multiple chemotherapy cycles may further reduce bone marrow reserve (Zhang et al., 2022). In contrast, the core mechanism of trilaciclib lies in its protection of the bone marrow hematopoietic system. By inhibiting cyclin-dependent kinase 4/6 (CDK4/6) and blocking the G1/S transition of the cell cycle, trilaciclib protects hematopoietic stem and progenitor cells from chemotherapy-induced damage, reduces the occurrence of chemotherapy-induced myelosuppression, and preserves bone marrow hematopoietic function (Tan et al., 2024; Goldschmidt et al., 2022). After the completion of chemotherapy cycles, trilaciclib is metabolically eliminated, the cell cycle switch is reactivated, and hematopoietic stem cells can rapidly resume normal proliferation, accelerating the recovery of neutrophils and platelets (Li et al., 2022). Furthermore, trilaciclib exerts immunomodulatory effects. In clinical studies of patients with SCLC and mTNBC, administration of trilaciclib before chemotherapy promoted the expansion of peripheral T cell clones, enhanced the activation level of CD8^+^ T cells, and strengthened the antitumor immune response (Daniel et al., 2021; Tan et al., 2023). However, these mechanistic explanations remain largely indirect and hypothesis-generating, as most included trials were not designed to directly evaluate immunologic or dose-intensity–related endpoints.

Our study found that trilaciclib administration significantly reduced the proportion of therapeutic use of ESAs (OR = 0.43, 95% CI = 0.22–0.81), G-CSF (OR = 0.33, 95% CI = 0.23–0.46), and RBC transfusion (OR = 0.56, 95% CI = 0.35–0.91). This finding is highly consistent with the results of a meta-analysis by Qiu et al. (2023), further confirming the reliability of trilaciclib in preventing myelosuppression. Notably, ESAs, G-CSF, and RBC transfusion all have definite risks of adverse reactions in clinical practice. A reduction in their use proportion can directly mitigate the impact of related adverse events on patients: Common adverse reactions of ESA administration include headache, generalized or local pain; in severe cases, it may induce cardiovascular events, hypertensive crisis, or even organ damage due to significant blood pressure elevation (Moradi et al., 2020; Rao et al., 2021). For G-CSF administration, bone pain is a common adverse reaction, occurring in approximately 10%–30% of patients with mild to moderate severity (Bumbăcea et al., 2024); severe cases may lead to splenic rupture, which carries a risk of death (Pagano et al., 2018), as well as severe allergic reactions. RBC transfusion is frequently associated with febrile non-hemolytic reactions and allergic reactions, mostly in adult patients. Moreover, long-term or massive transfusion may result in iron overload, as well as inflammation and thrombosis risks related to blood storage lesions. Clinically, it is necessary to additionally assess the transfusion necessity and blood storage duration in critically ill patients (Sharma et al., 2011; Hod et al., 2017). Thus, by reducing the demand for ESAs, G-CSF, and RBC transfusion, trilaciclib can not only alleviate the physiological burden of patients from therapeutic procedures but also decrease the risk of the aforementioned adverse reactions, while relieving patients’ psychological anxiety about treatment-related risks.

Notably, although a study by Qiu et al. has confirmed that there were no significant differences in OS and PFS between the trilaciclib group and the control group, with no negative impact on clinical chemotherapy outcomes observed, our study results exhibit a key discrepancy from this conclusion—patients receiving trilaciclib had significantly superior PFS compared to the control group. Although a statistically significant improvement in PFS was observed, this finding should be interpreted with caution. It is important to note that most of the included randomized trials were not primarily designed or powered to detect survival outcomes, and the duration of follow-up was relatively limited. Therefore, the observed PFS benefit should be considered exploratory and hypothesis-generating rather than definitive evidence of a survival advantage.

This discrepancy further suggests that trilaciclib may indirectly enhance chemotherapy efficacy through a dual mechanism: On one hand, trilaciclib protects bone marrow hematopoietic stem cells, reducing chemotherapy dose reductions caused by myelosuppression. Maintaining full chemotherapy doses is crucial for sustaining antitumor intensity, thereby indirectly contributing to improved efficacy. On the other hand, existing studies have verified that during chemotherapy, trilaciclib can promote the expansion of peripheral T cell clones and the formation of memory T cells, enhancing the immune system’s tumor surveillance capacity. This immune-enhancing effect is considered a potential driver of PFS prolongation (Stevens et al., 2021). Nevertheless, the consistency of the PFS direction across studies and the biological plausibility related to reduced chemotherapy dose delays and preserved immune function support the potential relevance of this finding. The reasons for the discrepancy between our study and Qiu et al.’s findings can be explained in two aspects: First, the difference in the scale of included studies—Qiu et al. included only 4 RCTs, while our study expanded to 6 RCTs. The larger sample size improved statistical power, making it easier to detect potential efficacy differences. Second, superior methodological rigor—on the basis of pooled analysis, our study added subgroup analysis to accurately identify factors influencing trilaciclib’s efficacy. Results showed that patients aged >65 years derived more significant benefits from trilaciclib treatment. These differences not only supplement evidence regarding the potential PFS benefits of trilaciclib but also provide more refined evidence support for personalized medication.

Subgroup analysis results showed that patients ≥65 years achieved greater benefits (HR = 0.54, 95% CI = 0.34–0.84), while no significant difference was observed in patients <65 years (HR = 0.83, 95% CI = 0.56–1.22). However, these subgroup analyses were limited by sample size and should be interpreted as exploratory. This observation may be partly explained by age-related physiological characteristics that could increase susceptibility to chemotherapy-induced myelosuppression in older patients. Elderly individuals have decreased hematopoietic stem cell reserve and regenerative capacity, making them more prone to chemotherapy-related hematological toxicities such as neutropenia, anemia, and thrombocytopenia. In a real-world multicenter study, the chemotherapy dose reduction rate was 11.3% in patients who received trilaciclib, compared with a significant 42.3% in historical controls without trilaciclib (Goldschmidt et al., 2023). Another study indicated that the chemotherapy dose reduction rate in patients ≥65 years was significantly lower than that in the placebo group, with a greater relative reduction than in patients <65 years (Hussein et al., 2021). Dose reductions lead to decreased chemotherapy intensity, which in turn shortens PFS. By reducing the dose reduction rate, trilaciclib enables elderly patients to receive standard-dose chemotherapy as planned, thereby improving efficacy.

Despite the favorable hematologic outcomes observed in this meta-analysis, several limitations should be acknowledged. First, substantial clinical heterogeneity was present across included studies, including differences in tumor types, chemotherapy regimens, dosing schedules, and timing of trilaciclib administration. Although random-effects models and sensitivity analyses were applied, residual heterogeneity may still influence the pooled estimates. Second, not all outcomes demonstrated consistent benefits. While reductions in severe neutropenia and supportive care use were robust across analyses, the effects on certain safety outcomes, such as leukopenia and diarrhea, were sensitive to the exclusion of individual studies, indicating that these findings should be interpreted with caution. Third, several included trials exhibited methodological limitations, particularly related to blinding and sample size, which may reduce the overall certainty of evidence. Although sensitivity analyses excluding studies with higher risk of bias did not materially alter most primary hematologic outcomes, these limitations may partially contribute to the observed variability across outcomes. Overall, while trilaciclib demonstrates consistent hematologic protective effects, its broader clinical benefits warrant further confirmation in large, well-designed trials.

## 5 Conclusion

Through a meta-analysis of six international multicenter RCTs, this study confirms that trilaciclib can effectively prevent chemotherapy-related myelosuppression: it significantly reduces the incidences of severe neutropenia (SN) and febrile neutropenia (FN), shortens the duration of SN, decreases the demand for ESAs, G-CSF, and RBC transfusion, without increasing the risk of adverse reactions such as anemia, nausea, vomiting, and fatigue. Meanwhile, trilaciclib can significantly prolong patients’ progression-free survival (PFS) but shows no significant improvement in overall survival (OS). Notably, patients ≥65 years and the American population derive more prominent benefits. The results of our study provide certain reference evidence for the use of trilaciclib in preventing chemotherapy-related myelosuppression in patients with solid tumors. However, more in-depth studies are still needed to fully confirm the efficacy and safety of trilaciclib.

## Data Availability

The original contributions presented in the study are included in the article/Supplementary Material, further inquiries can be directed to the corresponding authors.

## Supplementary material

SUPPLEMENTARY FIGURE S1 Forest plots of hazard ratio (HR) for outcomes. **(A)** Overall Survival (OS). **(B)** Progression-Free Survival (PFS). Analyzed using the inverse variance (IV) fixed-effect model.

SUPPLEMENTARY FIGURE S2 Subgroup analysis forest plots for Progression-Free Survival (PFS). **(A)** Age subgroup. **(B)** Race subgroup, both analyzed using the inverse variance (IV) fixed-effect model.

SUPPLEMENTARY FIGURE S3 Sensitivity analysis forest plots. **(A)** Severe neutropenia. **(B)** Granulocyte Colony-Stimulating Factor (G-CSF) infusion. **(C)** Leukopenia. **(D)** Overall Survival (OS). Analyzed using inverse variance (IV) fixed-effect model.

SUPPLEMENTARY FIGURE S4 Sensitivity analysis forest plots. **(A)** Nausea. **(B)** Diarrhea. **(C)** Fatigue. Analyzed using the Mantel-Haenszel (M-H) fixed-effect model.
