# Supplementary figures and images for: Trilaciclib for prophylaxis of chemotherapy-induced myelosuppression in solid tumor patients: a systematic review and meta-analysis
Source: Front Pharmacol. 2026 Apr 8;17:1754502. doi: 10.3389/fphar.2026.1754502 (PMC13100468; doi:10.3389/fphar.2026.1754502)

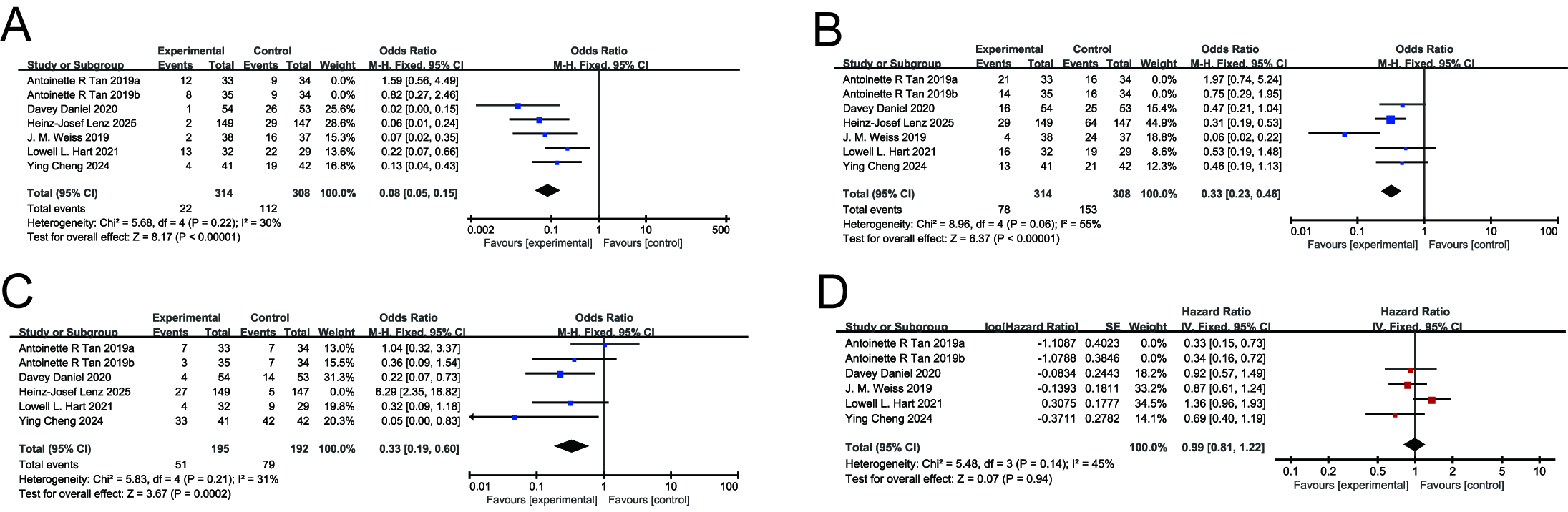

Supplement: Supplementary file 1 [file Image3.tif]

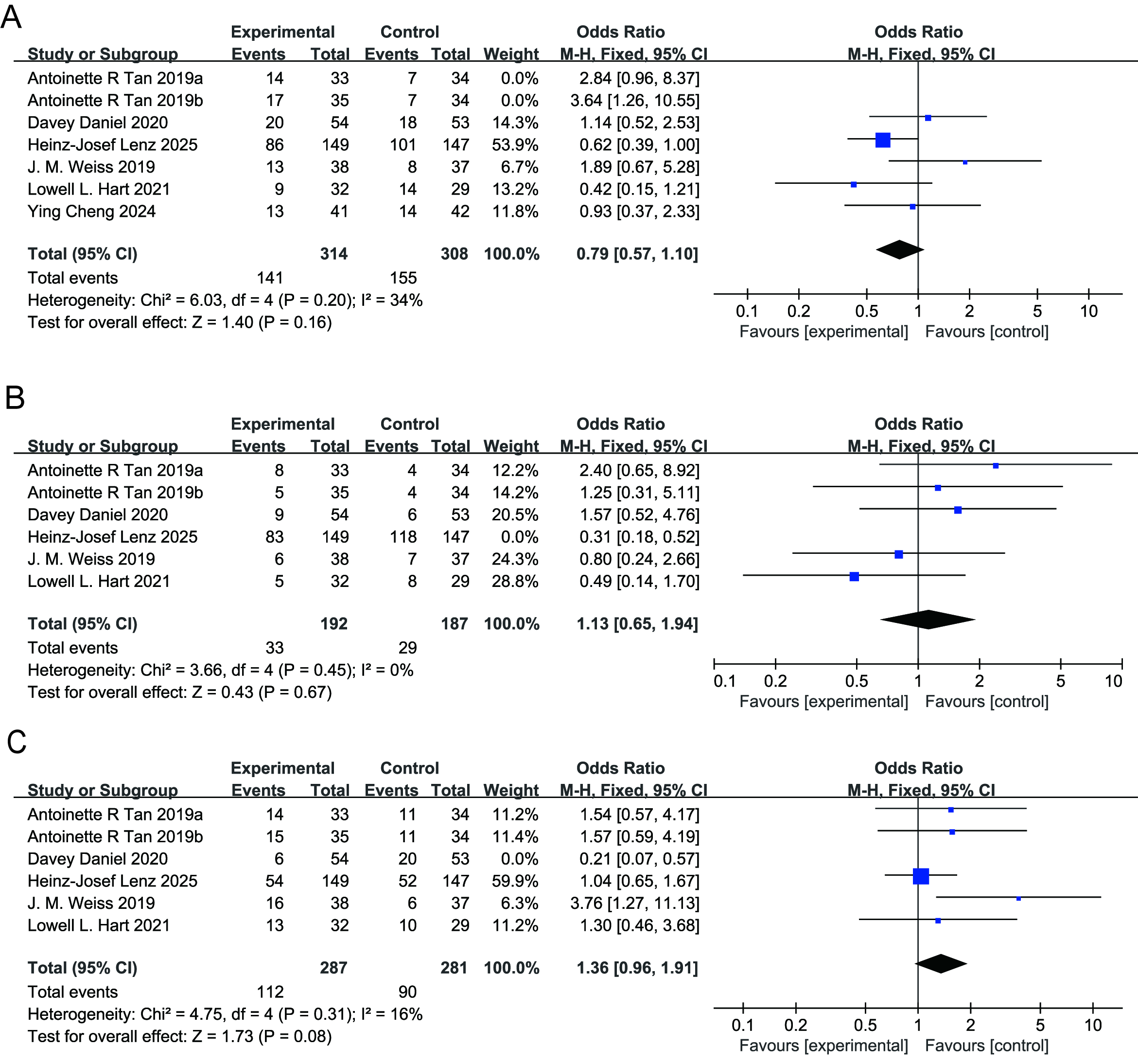

Supplement: Supplementary file 2 [file Image4.tif]

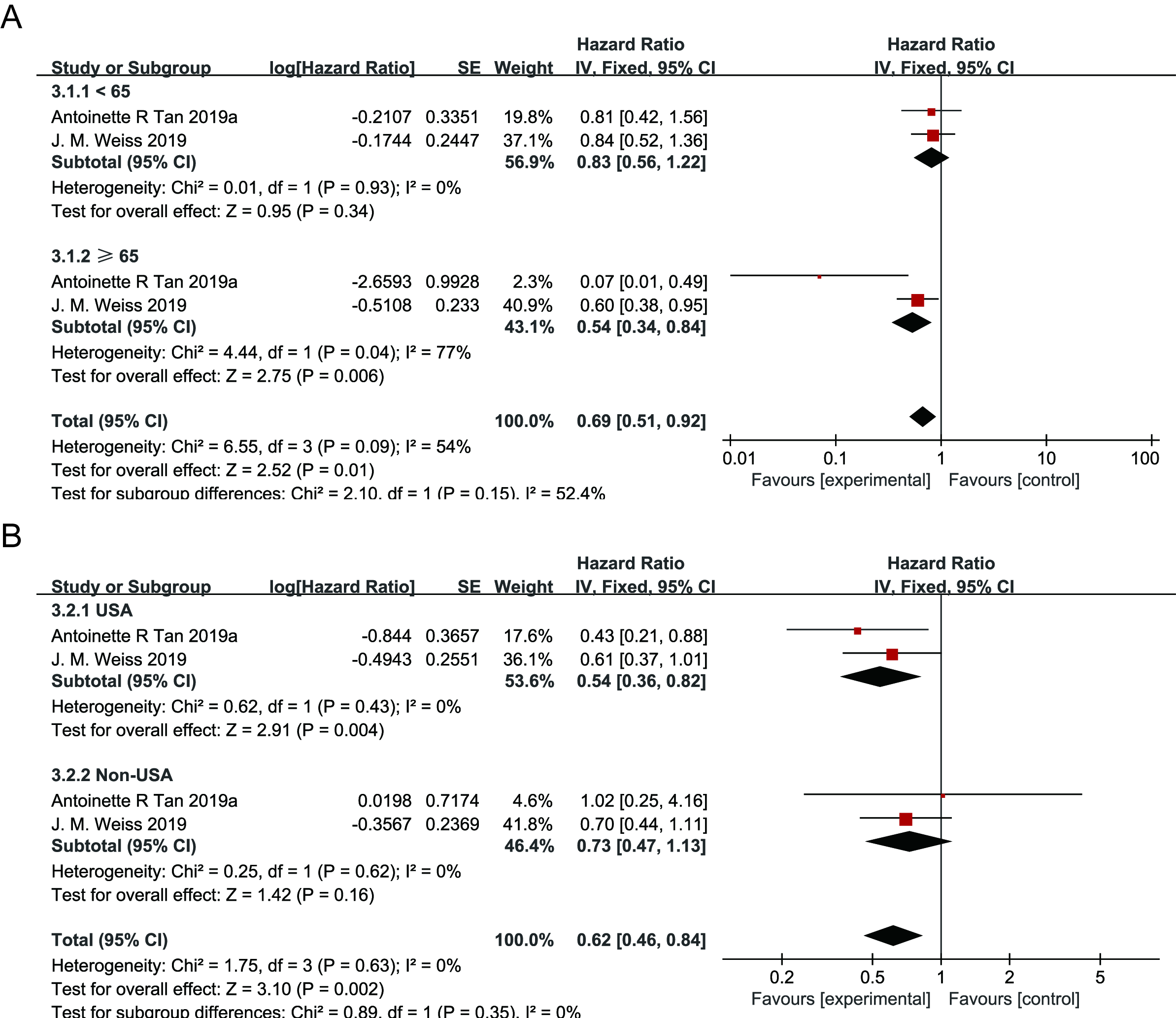

Supplement: Supplementary file 3 [file Image2.tif]

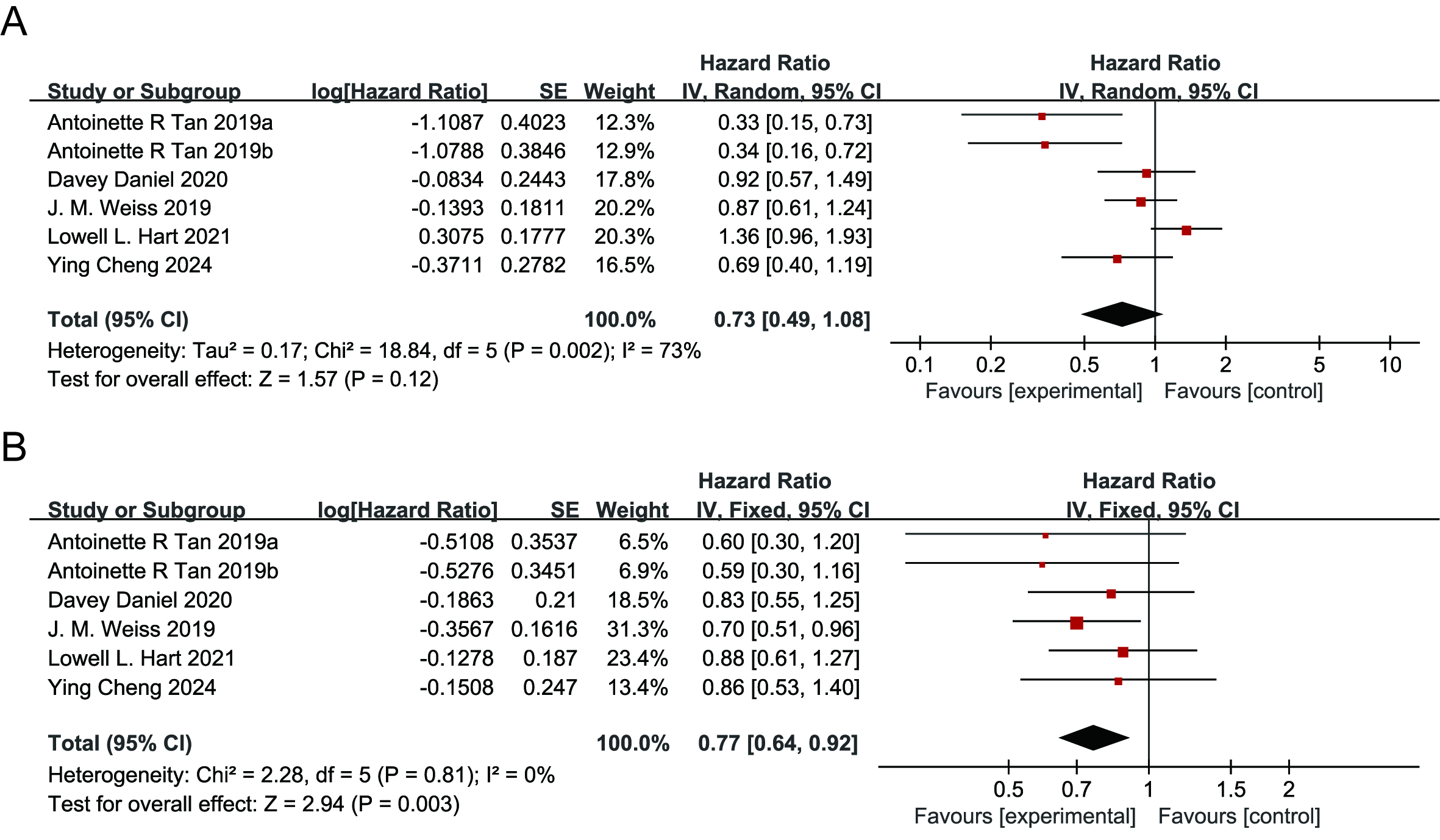

Supplement: Supplementary file 4 [file Image1.tif]
